# Review of Recent Treatment Strategies for Lumbar Disc Herniation (LDH) Focusing on Nonsurgical and Regenerative Therapies

**DOI:** 10.3390/jcm14041196

**Published:** 2025-02-12

**Authors:** Jae Sun Lee, Soo-Bin Lee, Kyung-Yil Kang, Seong Ho Oh, Dong-Sik Chae

**Affiliations:** 1 Department of Research Support Office of Medical & Sciences Research Institute, International St. Mary’s Hospital, Incheon 22711, Republic of Korea; vigorjaesun@hanmail.net; 2 Department of Orthopedic Surgery, Catholic Kwandong University, College of Medicine, International St. Mary’s Hospital, Incheon 22711, Republic of Korea; sumanzzz@ish.ac.kr (S.-B.L.); fbdlxk@naver.com (K.-Y.K.); ohho1201@naver.com (S.H.O.); 3 College of Medicine, Catholic Kwandong Graduate School, Gangneung-si 25601, Republic of Korea

**Keywords:** lumbar disc herniation, intervertebral discs degeneration, LIPUS, biomaterials, PRP, BMAC

## Abstract

Conservative treatment is primarily performed for the treatment of patients with lumbar disc herniation (LDH), but if it does not respond, surgical treatment can be performed. Surgical intervention has a positive effect on the rapid improvement of LDH symptoms. However, the effectiveness of surgical versus conservative treatment for LDH is controversial, especially regarding long-term effects. Recently, a treatment using platelet-rich plasma (PRP), bone marrow aspirate concentrate (BMAC), low-intensity pulsed ultrasound (LIPUS), etc., has been actively conducted as a treatment to avoid side effects of surgery and promote tissue regeneration. In this paper, the literature evaluating the effectiveness of non-surgical treatment options is reviewed with an emphasis on the effectiveness of clinical application. Several clinical studies have shown that PRP, biomaterials, BMAC, and LIPUS treatment promote tissue regeneration and alleviate symptoms. Although PRP-applied studies have suggested disc height changes, cell therapy and LIPUS treatment have many shortcomings in clinical aspects of tissue regeneration. Therefore, it is necessary to establish a unified, safe protocol and standardize the method of presenting results to confirm the clinical effect of the treatment for impaired intervertebral regeneration in patients with intervertebral disc degeneration (IDD), including LDH.

## 1. Introduction

Nearly 80% of the population experiences low back pain (LBP) at least once during their lifetime [1]. Lumbar disc herniation (LDH) is one of the most common causes of LBP affecting 1–3% of the general population and 43% of those under certain working conditions [2,3]. The highest prevalence of LDH occurs in individuals aged 30–50 years, with a male-to-female ratio of 2:1 [3,4]. LDH is not only painful but also financially burdensome to patients. It is estimated that the annual cost exceeds USD 100 billion in the USA [5], and the total cost for surgical intervention is USD 27,273, and USD 13,135 for conservative treatment per person [2].

The intervertebral discs (IVD) are important for maintaining normal spinal function. It consists of an inner nucleus pulposus (NP), outer annulus fibrosus (AF), and cartilaginous endplates that anchor the discs to adjacent vertebrae. The central NP secretes collagen and contains numerous proteoglycans (PG) that facilitate water retention and create hydrostatic pressure to resist axial compression of the spine [5]. Disc herniation occurs when the inner NP ruptures away from the annulus. The fragments of the disc material can then press on nerve roots located just behind the disc space. This can cause pain, weakness, numbness, or changes in sensation. Several risk factors, such as low water retention in the NP, degradation of collagen and extracellular matrix (ECM) materials, and inflammatory pathways, contribute to LDH [5]. Recent studies report that hereditary factors are the primary causes of LDH, especially for adolescent and young adult patients, and it is estimated that the condition has approximately 75% heredity origin [5,6,7].

Different treatments are available for patients with LDH. It can range from conservative to surgical therapy. When the disease is symptomatic, conservative treatment, including behavioural therapy, physical therapy, non-steroidal anti-inflammatory drugs (NSAIDs), oral steroids, and epidural steroid injections, is usually the first-line treatment [2]. If these treatments are not sufficient to alleviate symptoms, surgical intervention is usually recommended [8]. However, the outcomes of surgical interventions are not always superior to nonoperative treatment [8]. Although surgical treatment results in faster relief and marked improvement of symptoms, its long-term effects remain controversial [9,10]. Intraoperative nerve-related complications are common, and numbness and pain after surgery due to irritation of the nerve structures may occur [11].

Recently, in an attempt to avoid the possible side effects of surgery and promote tissue regeneration, studies on the safety and efficacy of applying physiochemically active substances such as PRP, cell therapy using BMAC, and LIPUS treatment for LDH have been actively attempted [11,12,13,14]. The purpose of this article was to review the current literature assessing the effectiveness of nonsurgical treatment options, including conservative care, application of PRP or BMAC, augmentation of biomaterials, and LIPUS therapy, with a focus on their clinical application and effectiveness.

## 2. Conservative Treatment

**Figure 1:**
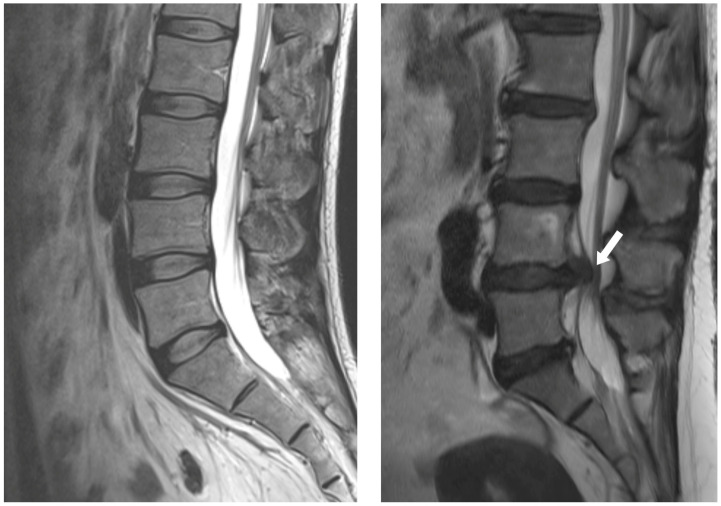
MRI images of a normal disc (**left**) and a herniated disc (white arrow) (**right**).

Most disc herniations occur in the lower lumbar spine, especially between the fourth and fifth lumbar vertebrae and between the fifth lumbar vertebra and the first sacral vertebra (L4-5 and L5-S1 levels) (Figure 1) [1]. In general, the first-line treatment for acute LDH is conservative therapy, which is prescribed for at least 6 weeks [15]. For nonsurgical management of LDH, NSAIDs, systemic steroids, opioids, anticonvulsants, and antidepressants are currently used to reduce pain [16]. Bed rest, an active lifestyle, and physical therapy also facilitate recovery from LDH [15]. More recently, new methods such as percutaneous ozone injection therapy have been developed with considerable improvements [17]. Most patients recover from LDH with conservative treatment without surgery [18]. However, surgery is more effective for symptom relief in cases where the patient is refractory to initial conservative treatment [19]. Predictors of failure in prolonged conservative treatment include male gender, low educational level, and intense and generalized pain [20].

There are several surgical techniques to treat LDH, such as open discectomy, endoscopic discectomy, micro-endoscopic discectomy, percutaneous endoscopic lumbar discectomy (PELD), percutaneous endoscopic interlaminar discectomy, fenestration discectomy, etc. More recently, full-endoscopic discectomy has allowed for minimally invasive access to the spinal canal using full-endoscopic visualization via the transforaminal and interlaminar corridors [21]. Minimally invasive techniques are gaining popularity in surgery, and micro-endoscopic discectomy is seen as one of the best methods for disc removal [22]. Although complications are uncommon, they should be acknowledged. A recent systematic review discussed that transforaminal endoscopic discectomy (TFED) is the best option for recurrent lumbar disc herniation (rLDH), which remains a challenge in spinal surgery [23]. TFED resulted in short surgical durations, minimal to no blood loss, and very low complication rates [23].

Surgical intervention has a positive effect on the rapid improvement of LDH symptoms. However, the effectiveness of surgical versus conservative treatment for LDH is controversial, especially regarding long-term effects. Researchers have compared the effectiveness of surgical and conservative treatments for LDH using various study designs, including prospective or retrospective cohort studies, randomized controlled trials (RCT), and systematic reviews.

Gugliotta et al. presented a prospective cohort study of a routine clinical practice registry of 370 patients and compared the short- and long-term effectiveness of surgical and conservative treatments for sciatica symptom severity. The pain score for non-surgical treatment was 5.3 at 6 weeks, and at 2 years of follow-up, it was 4.5, similar to 4.4 for surgical treatment. Regarding the physical function score, the 6-week value for non-surgical treatment was 36.3, and after 2 years, it was 42.8, similar to the value for surgical treatment [10]. In this study, they found faster pain relief after 3 months and less physical impairment at the 1-year follow-up in the surgical group. However, the difference between the groups was no longer present after that period [10]. A different result was observed in a single-centre randomized trial with 128 enrolled patients [24]. They found that patients who underwent surgery for sciatica lasting 4 to 12 months experienced a greater reduction in pain at 6 months than those who received conservative treatment [24]. At the 6-month follow-up, the leg pain intensity score was significantly lower in the surgical group (2.8) compared to the nonsurgical group (5.2), with an adjusted mean difference of 2.4 (95% confidence interval, 1.4 to 3.4; *p* < 0.001). Additionally, secondary outcomes, including the Oswestry Disability Index score and pain levels at 12 months, followed a similar positive trend in favour of the surgical group [24]. A systematic review with a sample of 2272 patients showed improved visual analogue scale (VAS) results in patients who underwent surgery within 1 to 3 months and 3 to 6 months, but the results were similar in the long term [25].

Patients judged as surgical candidates were also reported to have recovered after conservative treatment alone. Kim et al. conducted a prospective comprehensive cohort study to assess the outcomes of nonsurgical treatment for surgical candidates who requested nonsurgical management. In their study, the non-surgery group showed less improvement in the VAS scores at 1 month; however, no difference was observed between the groups after 24 months. The results demonstrate that even for surgical candidates, nonsurgical management may be a suitable option [26].

Conservative treatment is the recommended initial treatment for all cases of disc herniation because it allows for an early return to daily activities [20]. Patients deemed suitable for surgical treatment also recovered after conservative treatment. When choosing between different treatment options, especially when considering surgical intervention, the patient’s expectations and wishes must be considered, as with all treatments [20,26]. Patients who want relief from severe pain or who do not experience satisfactory outcomes with conservative treatment are more suitable for surgical treatment. However, for patients who are afraid of surgery or older adults with comorbidities, conservative treatment is better than surgical treatment.

Active search for and application of more conservative treatment methods will help expand the boundaries of conservative treatment and identify more efficient treatment methods. Recently, ozone therapy has been recognized for its safety and clinical efficacy in musculoskeletal disorders, including LBP, LDH, degenerative spinal disease, knee osteoarthritis, and meniscal injuries. Since the antibacterial effect of ozone was used to treat wounds during World War I, ozone treatment has been continuously studied in the medical field. Research has suggested relevant medical features of ozone, including its bactericidal and virucidal properties, inflammatory modulation, and circulatory stimulation [27]. Medical ozone therapy for LDH involves injecting a mixture of oxygen–ozone (O_2_-O_3_) generated by special devices directly or indirectly through several ways: subcutaneous, intramuscular, intraarticular, and intradiscal [28]. At concentrations that show no cytotoxicity, moderate oxidative stress produced by the interaction of biological components with O_3_ may exert positive effects on several tissues [27]. In a multicenter, double-blinded RCT involving 60 patients with LBP due to LDH, a significant difference was found between the O_2_-O_3_ injection treatment and the placebo groups in the percentage of patients who became pain-free (61% vs. 33%) after 6 months [29]. A recent study by Özcan et al. demonstrated the pre-and post-treatment pain scores in 62 patients with LBP. The authors reported a statistically significant improvement in the VAS and Oswestry Disability Index (ODI) scores at the 3-month follow-up [30].

## 3. Regenerative Therapies

Most current management strategies for spinal diseases aim for symptomatic relief and muscular stabilization without targeting IVD regeneration [31,32]. However, recent progress in regenerative medicine and tissue engineering technology offers the capability of cell- and biomaterial-based disc regeneration treatments. Current promising strategies for IVD regeneration include molecular therapy, which involves the injection of physiochemically active substances such as PRP; cell-based therapy utilizing mesenchymal stem cells (MSCs); and augmentation with biomaterials [33]. LIPUS has also been studied, focusing on bone enhancement or soft tissue regeneration. It has been reported that LIPUS treatment is effective in tendon, ligament, and bone-soft tissue healing in preclinical studies using animal models [34]. Since the capacity for IVD regeneration is based on the severity of disc degeneration, strategies for IVD regeneration treatment are based on the degree of IVD degeneration (IDD) [33]. For example, biomaterial-based therapies that maintain disc structure and activate the remaining disc cells are expected to be effective in LDH, while cell-based therapy for LDH with lumbar spinal canal stenosis, where the number of remaining cells is further reduced, is a better strategy [32]. Furthermore, the proper indications and timing of their introduction for these relatively new therapies should be fully discussed with patients [32]. Here, we review recent clinical cases of regenerative therapies applied to the treatment of LDH.

### 3.1. PRP Injection

The most widely studied molecular therapy for IVD regeneration in the clinical setting is the application of PRP because of its anabolic potential [33]. PRP contains many cytokines and growth factors, which are vascular endothelial growth factor (VEGF), interleukin-1 receptor antagonist, transforming growth factor-β1, platelet-derived growth factor, and insulin-like growth factor-1, etc., [35,36]. Due to its autologous and antimicrobial nature, PRP application can be considered to have minimal risks in immunogenic reactions, side effects, and surgical site infections [37].

Xu et al. [36] compared the safety and efficacy of the transforaminal injection of PRP with steroids in 124 patients with radicular pain due to LDH. In their prospective, randomized controlled study, the PRP and steroid groups did not show intergroup differences during follow-up over 1 year in the VAS, pressure pain thresholds, ODI, physical function, and bodily pain, suggesting the possible application of PRP injection as a safer alternative [36]. Another RCT using an epidural injection of PRP in 15 patients demonstrated a statistically and clinically significant reduction in LegVAS and ODI, which was comparable to the results obtained using triamcinolone [37]. Le et al. (2023) injected 4 mL of autologous PRP into the area of the affected nerve root of patients with radicular pain due to LDH and observed improvement in all three evaluation tools (VAS, ODI, and SLRT) at a 12-month follow-up with no associated complications [38].

Several meta-analyses have consistently concluded that PRP is safe and effective for treating LDH. A meta-analysis of PRP for LBP demonstrated a low incidence of adverse events compared to similar spinal injection techniques, with a well-documented safety profile [39]. Another meta-analysis of RCTs involving 131 patients with LBP showed that PRP injection significantly reduced pain scores and improved patient satisfaction with no increase in adverse events compared to the control intervention [40]. The timing of the pain-reducing effects of PRP injections remains controversial. Chang and Park (2021) analyzed three articles, including one RCT, concluding that intradiscal PRP injections showed that the pain-reducing effect significantly manifests two or six months after the injections but not after one month [41]. A long-term follow-up study reported that patients who received intradiscal PRP injections demonstrated statistically and clinically significant improvements in pain and function at 5–9 years [42].

Some studies combined PRP injections with surgical treatments. Jiang et al. (2022) carried out a prospective cohort study using transforaminal endoscopic lumbar discectomy (TELD) with and without PRP injection for LDH [11]. After the TELD procedure, the annular defect is the main reason for recurrent herniation. The study evaluated the effectiveness, disc remodelling, and recurrent rate of LDH in TELD with and without PRP in LDH treatment. The data showed that TELD with PRP injection was a safe and effective treatment for patients with LDH in the medium- and long-term follow-up, and that PRP injection was beneficial for disc remodelling and decreased the recurrence rate of LDH [11]. MRI observations during follow-up revealed a smaller spinal cross-sectional area and disc protrusion, indicating remodelling of the AF [11]. A combination of PELD and PRP hydrogel injection was performed in 98 consecutive patients with LDH in a retrospective study [43]. In this study, PRP injection was promising for disc remodelling during an 18-month follow-up period. The study evaluated the Pfirrmann grade, which shows changes in the structural organization of the IVD, and a significant difference at the final 18-month follow-up was found between the two groups [43].

As mentioned earlier, PRP contains numerous growth factors, which have physiological effects, including survival, growth, and differentiation of neurons, as well as angiogenesis, neurogenesis, and proliferation of mesenchymal cells [35]. Research on PRP treatment for LDH has demonstrated disc remodelling and nerve regeneration [11,43]. PRP has a proliferative effect on chondrocyte-like cells in the anterior medial AF, which can increase ECM production [11]. The most important point in using PRP is to increase the concentration of platelets at the targeted sites so that the released cytokines and GFs consequently regulate inflammation and immunological responses during tissue healing [44]. Studies have demonstrated that PRP, when combined with minimally invasive surgical techniques like percutaneous endoscopic lumbar discectomy, can significantly improve clinical outcomes and imaging results. PRP injections have been found to delay disc degeneration and enhance disc height, leading to better overall recovery [45]. PRP contains a high concentration of growth factors that can stimulate nerve tissue healing and regeneration. Studies have indicated that PRP can enhance axonal growth, provide neuroprotection, and improve blood supply to damaged nerve tissues, which are crucial for nerve repair and functional recovery [46]. It can be concluded that injection of autologous PRP can be considered an alternative therapeutic option to epidural steroids and surgery for the treatment of LDH [38]. However, large-scale, multicenter studies involving diverse patient populations are required to reinforce the current clinical evidence [39]. In addition, further intensive research is needed to develop an optimal protocol for PRP treatment and cost-effectiveness compared to conservative therapies, such as steroid application [36].

### 3.2. Cell-Based Therapy

Among disc remodelling therapies, cell-based therapies using minimally invasive procedures have drawn attention owing to their regenerative potential. A cell therapy approach aims to resolve disc inflammation by inhibiting the abnormal secretion of cytokines and the proliferation and stimulation of native cells [47]. Disc cells, notochordal cells, and MSCs have therapeutic potential in treating IDD. Among them, autologous and allogeneic MSCs from BMAC are the most used cell sources for clinical trials because of their immunomodulatory function and multipotent capability. MSCs isolated from bone marrow and adipose tissue can differentiate into a phenotype similar to that of NP [48]. MSCs also secrete hundreds of mediators, cytokines, and signalling molecules to effectively modulate inflammatory responses and control the infiltration process, which finally leads to regeneration [49]. MSC transplantation has become a theoretically attractive therapy because of its potential to treat various musculoskeletal disorders [50].

It is difficult to find a case in which BMAC treatment was performed by targeting only LDH in a clinical setting. Rather, stem cell treatment using BMAC is often performed for IDD, a more associated cause of LDH or LBP. El-Kadiry et al. (2021) applied autologous BMAC injection therapy to 13 patients with LBP and showed clinical improvement over 12 months using pain and functionality questionnaires [51]. MRI scans demonstrated an 11.45% increase in disc height and a 4.66% increase in spinal canal space size without worsening disc quality [51]. A systematic review of 16 studies comprising 607 patients appraised the analgesic efficacy of BMAC for discogenic pain and anatomical changes in the disc assessed using MRI [52]. The study showed general improvements in pain and functional scores compared to baseline and objective improvements to a certain degree on spinal MRIs in some studies [52]. Another recent systematic review reported that MSC injections may be a feasible option for treating IDD during the first phase of degeneration [53]. In contrast, another systematic review took a conservative stance on the effectiveness of cell therapy by highlighting the lack of strong clinical evidence and questioning the safety, long-term complications, and cost-effectiveness of the procedure [45].

An autologous BMAC source is the best option because it minimizes the risk of immune rejection. However, allogeneic BMAC is more convenient than autologous BMAC because the intervention is simple and does not require invasive procedures. A possible limitation of allogeneic BMAC is host immune rejection; however, it can be tolerated because of its immunomodulatory nature [54]. In an RCT, allogeneic BMAC from five healthy donors were used to treat 24 patients with degenerative disc disease. The study confirmed that allogeneic MSC therapy may be a valid alternative for the treatment of degenerative disc disease due to a quick and significant improvement in algal functional indices and improved Pfirrmann grading in the test group [55].

The use of cell therapy for the treatment of IDD poses several challenges. The IVD establishes an adverse microenvironment for resident cells and delivers exogenous cells, which limits the effect of cell therapy. This is due to its unique structure and characteristics, including being largely avascular, hypoxic, having low pH, high osmotic pressure, and high mechanical load [56]. This detrimental IVD environment raises several questions regarding cell survival and efficacy in vivo [52]. Therefore, the selection and securing of sufficient quality cells with high proliferative and differentiation potential is very important [32]. In addition, unstandardized protocols, including the preparation and dosage of injectables, unclear mechanisms of action of the biologic, and variable endpoints and measurement of outcomes, make comparison of data between studies difficult [51]. We have previously reported that the current clinical applications of MSC therapy for musculoskeletal diseases, such as degenerative osteoarthritis, lack consistency and low homogeneity in terms of detailed treatment methods [52]. Her et al. (2022) mentioned the same opinions regarding heterogeneity and poor generalizability among studies utilizing cell therapy for the treatment of IDD [52]. Therefore, a standardized protocol for the entire procedure, including outcome measures and follow-up periods, is necessary to build strong evidence for the efficacy of cell therapy.

### 3.3. Biomaterial-Based Therapies

Biomaterials are used alone or in combination with physiologically active components or cells to treat IVD. Biomaterial-based therapies involve the augmentation of biodegradable materials to maintain the disc structure by improving the disc height and mechanical stability of the vertebral segments [57]. The implantation of biomaterials can remove the mechanical compression of the nerve root and reduce pain caused by herniated discs [58]. Although details regarding their repair mechanisms have not been accurately identified, soft biomaterials allow the remaining resident disc cells to migrate into the structure and provide a framework for regeneration by native cells [32,59]. Several in vivo studies have demonstrated that various soft biomaterials have the potential to regenerate the IVD tissue by supporting the survival and stimulation of the remaining disc cells and facilitating ECM production [32]. Biomaterials for IVD regeneration include natural polymers, such as chitosan, collagen, gelatin, alginate, and hyaluronic acid (HA), and synthetic polymers, such as polyethylene glycol (PEG), polyurethane, polylactic acid, and polyglycolic acid [60]. Most materials have been recommended for use in combination because this allows for the mixing of properties from each material, thereby improving biodegradability, biocompatibility, or biomechanical functionality [57]. Several in vivo studies using cell-free biomaterials for the treatment of IVDs are listed in Table 1. Very few human clinical trials have investigated the use of biomaterials either alone or as cell scaffolds or delivery vehicles for IVD regeneration. Yin et al. (2014) reported clinical trials in which fibrin sealant was injected into the IVDs of patients for the treatment of symptomatic lumbar internal disc disruption, with significant improvement in pain and function at the 24-month follow-up (Table 1) [61].

In many cases, a biomaterial is used as a scaffold to stably fix physiologically active biocomponents at the injected site. Bolus injections of PRP are rapidly eliminated from the body, requiring delivery for the continuous release of PRP to improve therapeutic efficacy [74]. The controlled release of physiologically active substances in PRP, which have therapeutic effects, can be maintained using such supporting materials. Nagae et al. (2007) compared the results obtained from groups treated with PRP-gelatin hydrogel microspheres, PRP alone, or PBS-impregnated microspheres [75]. The authors observed a decrease in the IDD rate with an increase in PG expression, significantly less severe lesions in the NP and AF, and an increase in IVD height in the group treated with PRP-gelatin-hydrogel-microspheres after two years [75,76]. Kumar et al. (2017) studied the safety and tolerability of intradiscal implantation of combined autologous adipose-derived MSC and HA derivatives in patients with chronic discogenic LBP [77]. The phase I clinical trial enrolled 10 patients with eligibility, and the 10 patients underwent a single intradiscal injection at different doses (2 × 10^7^ cells/disc, 4 × 10^7^ cells/disc). The authors reported significant improvements in VAS, ODI, and SF-36 scores in both groups, and three patients were found to have increased water content in the disc according to diffusion MRI, demonstrating the promise of this regenerative medicine approach [77].

In general, augmented biomaterials can reduce the compressive force on nerve roots and provide a more favourable environment for native cell proliferation and regeneration [57]. Another benefit of using biological materials as carriers for cells or physiologically active substances is the improved stability of bioactivity because they are protected against proteolytic and other denaturation processes after in vivo application [75]. Choi et al. showed that PRP remained active upon encapsulation and release from a PEG hydrogel for up to 14 days (until complete PEG gel degradation) [74]. The clinical application of biomaterial-based therapies requires further improvements in surgical techniques, in vivo stability, biodegradability, and non-immunogenicity [32,57].

### 3.4. Low-Intensity Pulsed Ultrasound (LIPUS)

LIPUS utilizes low-intensity pulsatory mechanical waves to provide non-invasive physical stimulation to target tissues. LIPUS has regenerative and anti-inflammatory effects; therefore, its therapeutic applications vary. In the field of orthopedics, LIPUS is an attractive treatment option for bone fractures, tendon and cartilage recovery, and IDD. Studies have shown that the effect of LIPUS in treating disease-associated tissues may be primarily associated with the upregulation of cell proliferation via activation of the integrin receptor and Rho/ROC/Src/ERK signalling pathway [78]. It also affects the expression of osteocalcin, alkaline phosphatase, VEGF, and MMP-13 expression [79]. In LDH, LIPUS likely functions by reabsorbing the escaped disc. Animal model experiments and in vitro studies have confirmed that reabsorption of the herniated disc occurs when LIPUS is used [80,81].

The IVD with weak vascularity is composed mainly of the AF, and the internal gelatinous vaginal nucleus is composed of chondrocytes. A recent MRI analysis of patients with IVD revealed that IVD can be reabsorbed spontaneously without surgery, and this reabsorption is associated with abundant vascular formation, macrophage infiltration, and upregulated MMP-3 expression [82]. In addition, IVD degeneration occurs due to changes in PG biosynthesis in the IVD. Therefore, the upregulation of PG synthesis in IVD cells may be an approach for treating disc degeneration. LIPUS promotes ECM synthesis in degenerative human NP cells by activating the FAK/PI3K/Akt pathway [83].

Few studies have demonstrated the clinical efficacy of LIPUS in the spinal region. A randomized controlled study examined the effects of LIPUS in patients with lumbar spondylolysis and observed significant pain reduction and functional disability, demonstrating that LIPUS is a safe and effective method for early bone healing [84]. Another clinical trial compared high-intensity laser therapy (HILT) with a combination of ultrasound treatment and transcutaneous nerve stimulation (TENS) for cervical pain associated with cervical disc herniation; no statistical significance was found between the two groups in terms of cervical range of motion, VAS, and neck pain and disability scale scores [85]. However, both groups showed significant improvements, indicating that ultrasound has the potential as a therapeutic option for IVD. Rubira et al. (2019) compared the effects of low-level laser and pulsed and continuous ultrasound on pain and physical disability in chronic nonspecific LBP in a randomized controlled clinical trial [86]. They divided 100 volunteers into four groups: pulsed laser, pulsed ultrasound, continuous ultrasound, and control. They observed pain reduction in all three intervention groups, with greater relative pain reduction in the pulsed laser group and the highest relative gain in functional disability in the pulsed ultrasound group [86]. Boyraz et al. (2015) compared HILT and ultrasound treatment in patients with lumbar discopathy and showed a statistically significant improvement in the third month, although there was no difference between the groups ten days after the intervention [87].

A more controlled experimental design is required to investigate the therapeutic effects of LIPUS on LDH levels. Current studies have combined and compared various treatments rather than using LIPUS alone. In studies using methods such as exercise, hot packs, and TENS, it was difficult to measure the therapeutic effect of LIPUS alone. In addition, when comparing treatment effects, a radiological evaluation that can confirm tissue regeneration is often omitted. Therefore, it is currently difficult to confirm the tissue regeneration effect of LIPUS. Future studies are needed to determine the exact effect of LIPUS on IDD in a more controlled environment and standardization of protocols, with a focus on tissue regeneration.

## 4. Conclusions

When the symptoms of LDH are not relieved after conservative therapy, surgical treatment for rapid pain improvement and recovery is chosen; however, research examining the long-term effects of non-surgical treatment and surgical treatment reported no difference between the two groups. According to the findings of this review, patients who required surgical treatment also improved with non-surgical treatment. Considering the operative cost, possible adverse effects, and patients’ fear of the surgery, it seems necessary to take a more careful approach by integrating the observation of the patient’s condition and opinion before switching to surgical treatment. In addition, clinical trials for new treatment methods should be conducted. The non-surgical methods discussed in this review show a certain level of clinical efficacy in the treatment of IDD, including LDH. Further research should be conducted on therapies that can promote tissue regeneration in patients with IDD, including LDH. Based on clinical studies using PRP, biomaterials, BMAC, and LIPUS, we observed that each method promoted tissue regeneration and alleviated symptoms. According to the conclusions thus far, the actual height change in the disc is presented in a PRP application study; however, clinical studies showing solid evidence of tissue regeneration are limited in the case of cell therapy and LIPUS. Therefore, a unified and safe protocol is required to compare the regenerative effects of each treatment. The method for presenting the results should also be standardized so that the effectiveness of the treatment methods can be compared.

This review has a couple of limitations. This review may introduce subjectivity and bias, lacking a systematic methodology. The quality of included studies can vary, potentially leading to incomplete coverage of the literature.

## Figures and Tables

**Table 1:** In vivo studies used biomaterials (acellular only) for IVD treatment.

Biomaterials		Prepared Form	Model	Injury Type	Results	Ref.
Synthetic Biomaterials	PGA (polyglycolic acid)	PGA-hyaluronan implants in allogeneic serum	Rabbits	Disc defect (Discectomy)	Tissue regenerated and disc water content improved after 12 months.	[62]
	PLGA (polylactic-co-glycolic) acid	PLGA/fibrin gel plugs in homogeneous fibrinogen solution	Rabbits	Disc defect (Discectomy)	Significantly higher nerve fibres were observed than control.	[63]
Natural Biomaterials	Alginate	bioresorbable ultra-purified alginate (UPAL) gel in saline solution	Rabbits and sheep	Disc defect (Discectomy)	Endogenous NP progenitor cells propagated, and an extracellular matrix was produced.	[64]
	Alginate	Dry and fibrous UPAL gel in saline solution	Rats and rabbits	Disc defect (Discectomy)	Suppressed TNF-α and IL-6 production, downregulated TrkA expression, inhibited IVD degeneration, and reduced nociceptive behaviour observed.	[65]
	Fibrin	BIOSTAT BIOLOGX^®^ Fibrin Sealant (FS)(Spinal Restoraion, Inc., Austin, TX, USA)	Minipigs	Degenerative injury (nucleotomy)	Structural, compositional, and mechanical repair was facilitated.	[66]
	Fibrin	fibrin hydrogel crosslinked with genipin (FibGen)	Sheep	Biopsy-type AF defect	Both untreated and FibGen-treated groups showed equivalency with no detectable differences in various outcomes.	[67]
	Fibrin	BIOSTAT BIOLOGX^®^ FS	Human	Chronic, single, or contiguous two-level lumbar discogenic pain	Mean low back pain VAS (Visual Analogue Scale) scores decreased, and mean RMDQ (Roland Morris Disability Questionnaire) scores improved.	[61]
	Hyaluronic acid (HA)	HA hydrogel in phosphate-buffered saline (PBS)	Rats	Surgical puncture-induced disc injury	Nociceptive behaviour reduced, hyperinnervation inhibited, and inflammation and regulation of matrix components attenuated.	[68]
	HA	HA/collagen hydrogel matrix with in situ polymerization after implantation	Minipigs	Partial nucleotomy	HA treatment resulted in more scarring and inflammation in the annulus, and gene expression of catabolic MMPs was upregulated.	[69]
	Collagen	Mechanically stable collagen cryogel and fibrillated collagen with shape-memory	Rats	Tail nucleotomy	The treatment exhibited outstanding chondrogenic activities, alleviated mechanical allodynia, maintained a higher concentration of water content, and preserved the disc structure by restoring the matrix proteins.	[70]
	Collagen	Bioorthogonal groups conjugated onto gelatin to create injectable hydrogel w/TGFβ	Rats	Nucleotomy via aspiration	TGFβ-loaded hydrogel resulted in the greatest water retention and displayed substantial histological recovery.	[71]
Combination	polyethylene glycol diacrylate (PEGDA), genipin-crosslinked fibrin (FibGen),carboxymethyl cellulose–methylcellulose (C-MC)	Combining adhesive and nonadhesive injectable hydrogels	Sheep	Partial discectomy	All repaired IVDs maintained 90% of their preoperative disc height. C-MC and FibGen + C-MC groups had the best outcomes and may be appropriate for enhancement with bioactive factors.	[72]
	PGA, hyaluronan	Freeze-dried resorbable polyglycolic acid (PGA)–hyaluronan implants in autologous sheep serum	Sheep	Partial discectomy	Implantation induced nucleus pulposus tissue regeneration and improved disc water content.	[73]

## Data Availability

No new data were created or analyzed in this study. Data sharing is not applicable to this article.

## Abbreviations

The following abbreviations are used in this manuscript:

PRP	Platelet-rich plasma
BMAC	Bone marrow aspirate concentrate
MSC	Mesenchymal stem cells
LDH	Lumbar disc herniation
IDD	Intervertebral disc degeneration
LIPUS	Low-intensity pulsed ultrasound
HILT	High-intensity laser therapy
TEN	Transcutaneous nerve stimulation
VAS	Visual analogue scale
ROM	Range of motion

## References

[1] Al Qaraghli M.I., De Jesus O. (2023). Lumbar disc herniation. StatPearls.

[2] Patel S.A., Wilt Z., Gandhi S.D., Rihn J.A. (2016). Cost-effectiveness of treatments for lumbar disc herniation. Seminars in Spine Surgery.

[3] Zhang A.S., Xu A., Ansari K., Hardacker K., Anderson G., Alsoof D., Daniels A.H. (2023). Lumbar disc herniation: Diagnosis and management. Am. J. Med..

[4] Jordan J.L., Konstantinou K., O’Dowd J. (2011). Herniated lumbar disc. BMJ Clin. Evid..

[5] Amin R.M., Andrade N.S., Neuman B.J. (2017). Lumbar disc herniation. Curr. Rev. Musculoskelet. Med..

[6] Ravichandran D., Pillai J., Krishnamurthy K. (2022). Genetics of intervertebral disc disease: A review. Clin. Anat..

[7] Qi L., Luo L., Meng X., Zhang J., Yu T., Nie X., Liu Q. (2022). Risk factors for lumbar disc herniation in adolescents and young adults: A case–control study. Front. Surg..

[8] Awad J.N., Moskovich R. (2006). Lumbar disc herniations: Surgical versus nonsurgical treatment. Clin. Orthop. Relat. Res..

[9] Wan Z.Y., Shan H., Liu T.F., Song F., Zhang J., Liu Z.H., Ma K.L., Wang H.Q., Wang H.Q. (2022). Emerging issues questioning the current treatment strategies for lumbar disc herniation. Front. Surg..

[10] Gugliotta M., da Costa B.R., Dabis E., Theiler R., Jüni P., Reichenbach S., Landolt H., Hasler P., Hasler P. (2016). Surgical versus conservative treatment for lumbar disc herniation: A prospective cohort study. BMJ Open.

[11] Jiang Y., Zuo R., Yuan S., Li J., Liu C., Zhang J., Ma M., Li D., Hai Y., Li D. (2022). Transforaminal endoscopic lumbar discectomy with versus without platelet-rich plasma injection for lumbar disc herniation: A prospective cohort study. Pain. Res. Manag..

[12] Wirth F., Bergamaschi E.C.Q.A., Forti F.S., Bergamaschi J.P.M. (2023). Development of indications for endoscopic spine surgery: An overview. Int. J. Transl. Med..

[13] Ozen S., Guzel S., Senlikci H.B., Cosar S.N.S., Selcuk E.S. (2023). Efficacy of ultrasound versus short wave diathermy in the treatment of chronic low back pain in patients with lumbar disk herniation: A prospective randomized control study. BMC Sports Sci. Med. Rehabil..

[14] Ariel E., Levkovitz Y., Goor-Aryeh I., Motti R. (2022). The effects of TENS, interferential stimulation, and combined interferential stimulation and pulsed ultrasound on patients with disc herniation-induced radicular pain. J. Back. Musculoskelet. Rehabil..

[15] Altun I., Yüksel K.Z. (2017). Lumbar herniated disc: Spontaneous regression. Korean J. Pain..

[16] Rasi A.M., Mirbolook A., Darestani R.T., Sayadi S., Ebadi S.S. (2020). Conservative treatment of low back pain in lumbar disc herniation: Comparison of three therapeutic regimens. Syst. Rev. Pharm..

[17] Migliorini F., Maffulli N., Eschweiler J., Bestch M., Tingart M., Baroncini A. (2020). Ozone injection therapy for intervertebral disc herniation. Br. Med. Bull..

[18] Lilly D.T., Davison M.A., Eldridge C.M., Singh R., Montgomery E.Y., Bagley C., Adogwa O. (2021). An assessment of nonoperative management strategies in a herniated lumbar disc population: Successes versus failures. Glob. Spine J..

[19] Arts M.P., Kuršumović A., Miller L.E., Wolfs J.F.C., Perrin J.M., Van de Kelft E., Heidecke V. (2019). Comparison of treatments for lumbar disc herniation: Systematic review with network meta-analysis. Medicine.

[20] Alves Filho A.C., Gonçalves A.L.F., Barbosa A.M. (2021). Conservative versus surgical treatment in patients with lumbar disc herniation. Braz. J. Pain.

[21] Li W.S., Yan Q., Cong L. (2022). Comparison of endoscopic discectomy versus non-endoscopic discectomy for symptomatic lumbar disc herniation: A systematic review and meta-analysis. Glob. Spine J..

[22] Chang C.P., Lee W.S., Lee S.C. (2006). Left internal iliac artery and vein tear during microendoscopic lumbar discectomy—A case report. Minim. Invasive Ther. Allied Technol..

[23] Musa G., Abakirov M.D., Arzoumi N., Mamyrbaev S.T., Castillo R.E.B., Chmutin G.E., Montemurro N. (2025). Is Transforaminal Endoscopic Discectomy the Best Option for Recurrent Lumbar Disc Herniation? A Systematic Review. Int. J. Spine Surg..

[24] Bailey C.S., Rasoulinejad P., Taylor D., Sequeira K., Miller T., Watson J., Rosedale R., Bailey S.I., Gurr K.R., Siddiqi F. (2020). Surgery versus conservative care for persistent sciatica lasting 4 to 12 months. N. Engl. J. Med..

[25] Chen B.L., Guo J.B., Zhang H.W., Zhang Y.J., Zhu Y., Zhang J., Hu H.Y., Zheng Y.L., Wang X.Q. (2018). Surgical versus non-operative treatment for lumbar disc herniation: A systematic review and meta-analysis. Clin. Rehabil..

[26] Kim C.H., Choi Y., Chung C.K., Kim K.J., Shin D.A., Park Y.K., Kwon W.K., Yang S.H., Lee C.H., Park S.B. (2021). Nonsurgical treatment outcomes for surgical candidates with lumbar disc herniation: A comprehensive cohort study. Sci. Rep..

[27] de Sire A., Agostini F., Lippi L., Mangone M., Marchese S., Cisari C., Bernetti A., Invernizzi M., Invernizzi M. (2021). Oxygen–Ozone therapy in the rehabilitation field: State of the art on mechanisms of action, safety and effectiveness in patients with musculoskeletal disorders. Biomolecules.

[28] Akkawi I. (2020). Ozone therapy for musculoskeletal disorders: Current concepts. Acta Biomed..

[29] Paoloni M., Di Sante L., Cacchio A., Apuzzo D., Marotta S., Razzano M., Franzini M., Santilli V., Santilli V. (2009). Intramuscular oxygen-ozone therapy in the treatment of acute back pain with lumbar disc herniation: A multicenter, randomized, double-blind, clinical trial of active and simulated lumbar paravertebral injection. Spine (Phila. Pa. 1976).

[30] Özcan Ç., Polat Ö., Çelik H., Uçar B.Y. (2019). The effect of paravertebral ozone injection in the treatment of low back pain. Pain. Pract..

[31] Wei A., Shen B., Williams L., Diwan A. (2014). Mesenchymal stem cells: Potential application in intervertebral disc regeneration. Transl. Pediatr..

[32] Yamada K., Iwasaki N., Sudo H. (2022). Biomaterials and cell-based regenerative therapies for intervertebral disc degeneration with a focus on biological and biomechanical functional repair: Targeting treatments for disc herniation. Cells.

[33] Ju D.G., Kanim L.E., Bae H.W. (2020). Intervertebral disc repair: Current concepts. Glob. Spine J..

[34] Lai W.C., Iglesias B.C., Mark B.J., Wang D. (2021). Low-intensity pulsed ultrasound augments tendon, ligament, and bone–soft tissue healing in preclinical animal models: A systematic review. Arthroscopy.

[35] Rawson B. (2020). Platelet-rich plasma and epidural platelet lysate: Novel treatment for lumbar disk herniation. J. Am. Osteopath. Assoc..

[36] Xu Z., Wu S., Li X., Liu C., Fan S., Ma C. (2021). Ultrasound-guided transforaminal injections of platelet-rich plasma compared with steroid in lumbar disc herniation: A prospective, randomized, controlled study. Neural Plast..

[37] Wongjarupong A., Pairuchvej S., Laohapornsvan P., Kotheeranurak V., Jitpakdee K., Yeekian C., Chanplakorn P. (2023). ‘Platelet-Rich Plasma’ epidural injection an emerging strategy in lumbar disc herniation: A randomized controlled trial. BMC Musculoskelet. Disord..

[38] Le V.T., Nguyen Dao L.T.N., Nguyen A.M. (2023). Transforaminal injection of autologous platelet-rich plasma for lumbar disc herniation: A single-center prospective study in Vietnam. Asian J. Surg..

[39] Machado E.S., Soares F.P., Vianna de Abreu E., de Souza T.A.D.C., Meves R., Grohs H., Ambach M.A., Navani A., de Castro R.B., Pozza D.H. (2023). Systematic review of platelet-rich plasma for low back pain. Biomedicines.

[40] Xuan Z., Yu W., Dou Y., Wang T. (2020). Efficacy of platelet-rich plasma for low back pain: A systematic review and meta-analysis. J. Neurol. Surg. A Cent. Eur. Neurosurg..

[41] Chang M.C., Park D. (2021). The effect of intradiscal platelet-rich plasma injection for management of discogenic lower back pain: A meta-analysis. J. Pain. Res..

[42] Cheng J., Santiago K.A., Nguyen J.T., Solomon J.L., Lutz G.E. (2019). Treatment of symptomatic degenerative intervertebral discs with autologous platelet-rich plasma: Follow-up at 5–9 years. Regen. Med..

[43] Zhang L., Zhang C., Song D., Chen G., Liu L. (2023). Combination of percutaneous endoscopic lumbar discectomy and platelet-rich plasma hydrogel injection for the treatment of lumbar disc herniation. J. Orthop. Surg. Res..

[44] Bhatia R., Chopra G. (2016). Efficacy of platelet rich plasma via lumbar epidural route in chronic prolapsed intervertebral disc patients-a pilot study. J. Clin. Diagn. Res..

[45] Wang S., Liu X., Wang Y. (2022). Evaluation of platelet-rich plasma therapy for peripheral nerve regeneration: A critical review of literature. Front. Bioeng. Biotechnol..

[46] Li T., Du W., Ding Z., Liu J., Ding Y. (2024). Percutaneous endoscopic lumbar discectomy combined with platelet-rich plasma injection for lumbar disc herniation: Analysis of clinical and imaging outcomes. BMC Musculoskelet. Disord..

[47] Meisel H.J., Agarwal N., Hsieh P.C., Skelly A., Park J.B., Brodke D., Wang J.C., Yoon S.T., Buser Z. (2019). Cell therapy for treatment of intervertebral disc degeneration: A systematic review. Glob. Spine J..

[48] Clarke L.E., McConnell J.C., Sherratt M.J., Derby B., Richardson S.M., Hoyland J.A. (2014). Growth differentiation factor 6 and transforming growth factor-beta differentially mediate mesenchymal stem cell differentiation, composition, and micromechanical properties of nucleus pulposus constructs. Arthritis Res. Ther..

[49] Markov A., Thangavelu L., Aravindhan S., Zekiy A.O., Jarahian M., Chartrand M.S., Pathak Y., Marofi F., Shamlou S., Hassanzadeh A. (2021). Mesenchymal stem/stromal cells as a valuable source for the treatment of immune-mediated disorders. Stem Cell Res. Ther..

[50] Law L., Hunt C.L., Van Wijnen A.J., Nassr A., Larson A.N., Eldrige J.S., Mauck W.D., Pingree M.J., Yang J., Muir C.W. (2019). Office-based mesenchymal stem cell therapy for the treatment of musculoskeletal disease: A systematic review of recent human studies. Pain. Med..

[51] El-Kadiry A.E.H., Lumbao C., Rafei M., Shammaa R. (2021). Autologous BMAC therapy improves spinal degenerative joint disease in lower back pain patients. Front. Med..

[52] Her Y.F., Kubrova E., Martinez Alvarez G.A., D’Souza R.S. (2022). The analgesic efficacy of intradiscal injection of bone marrow aspirate concentrate and culture-expanded bone marrow mesenchymal stromal cells in discogenic pain: A systematic review. J. Pain. Res..

[53] Migliorini F., Rath B., Tingart M., Baroncini A., Quack V., Eschweiler J. (2019). Autogenic mesenchymal stem cells for intervertebral disc regeneration. Int. Orthop..

[54] Ryan J.M., Barry F.P., Murphy J.M., Mahon B.P. (2005). Mesenchymal stem cells avoid allogeneic rejection. J. Inflamm..

[55] Noriega D.C., Ardura F., Hernández-Ramajo R., Martín-Ferrero M.Á., Sánchez-Lite I., Toribio B., Alberca M., García V., Moraleda J.M., Sánchez A. (2017). Intervertebral disc repair by allogeneic mesenchymal bone marrow cells: A randomized controlled trial. Transplantation.

[56] Peng B., Li Y. (2022). Concerns about cell therapy for intervertebral disc degeneration. NPJ Regen. Med..

[57] Schutgens E.M., Tryfonidou M.A., Smit T.H., Öner F.C., Krouwels A., Ito K., Creemers L.B. (2015). Biomaterials for intervertebral disc regeneration: Past performance and possible future strategies. Eur. Cell Mater..

[58] DiZerega G.S., Traylor M.M., Alphonso L.S., Falcone S.J. (2010). Use of temporary implantable biomaterials to reduce leg pain and back pain in patients with sciatica and lumbar disc herniation. Materials.

[59] Anderson D.G., Risbud M.V., Shapiro I.M., Vaccaro A.R., Albert T.J. (2005). Cell-based therapy for disc repair. Spine J..

[60] Choi Y., Park M.H., Lee K. (2019). Tissue engineering strategies for intervertebral disc treatment using functional polymers. Polymers.

[61] Yin W., Pauza K., Olan W.J., Doerzbacher J.F., Thorne K.J. (2014). Intradiscal injection of fibrin sealant for the treatment of symptomatic lumbar internal disc disruption: Results of a prospective multicenter pilot study with 24-month follow-up. Pain. Med..

[62] Endres M., Abbushi A., Thomale U.W., Cabraja M., Kroppenstedt S.N., Morawietz L., Casalis P.A., Zenclussen M.L., Lemke A.J., Horn P. (2010). Intervertebral disc regeneration after implantation of a cell-free bioresorbable implant in a rabbit disc degeneration model. Biomaterials.

[63] Xin L., Xu W., Yu L., Fan S., Wang W., Yu F., Wang Z. (2017). Effects of annulus defects and implantation of poly(lactic-co-glycolic acid) (PLGA)/fibrin gel scaffolds on nerves ingrowth in a rabbit model of annular injury disc degeneration. J. Orthop. Surg. Res..

[64] Tsujimoto T., Sudo H., Todoh M., Yamada K., Iwasaki K., Ohnishi T., Hirohama N., Nonoyama T., Ukeba D., Ura K. (2018). An acellular bioresorbable ultra-purified alginate gel promotes intervertebral disc repair: A preclinical proof-of-concept study. EBiomedicine.

[65] Ura K., Yamada K., Tsujimoto T., Ukeba D., Iwasaki N., Sudo H. (2021). Ultra-purified alginate gel implantation decreases inflammatory cytokine levels, prevents intervertebral disc degeneration, and reduces acute pain after discectomy. Sci. Rep..

[66] Buser Z., Kuelling F., Liu J., Liebenberg E., Thorne K.J., Coughlin D., Lotz J.C. (2011). Biological and biomechanical effects of fibrin injection into porcine intervertebral discs. Spine.

[67] Long R.G., Ferguson S.J., Benneker L.M., Sakai D., Li Z., Pandit A., Grijpma D.W., Eglin D., Zeiter S., Schmid T. (2020). Morphological and biomechanical effects of annulus fibrosus injury and repair in an ovine cervical model. JOR Spine.

[68] Mohd Isa I.L., Abbah S.A., Kilcoyne M., Sakai D., Dockery P., Finn D.P., Pandit A. (2018). Implantation of hyaluronic acid hydrogel prevents the pain phenotype in a rat model of intervertebral disc injury. Sci. Adv..

[69] Omlor G.W., Nerlich A.G., Lorenz H., Bruckner T., Richter W., Pfeiffer M., Gühring T. (2012). Injection of a polymerized hyaluronic acid/collagen hydrogel matrix in an in vivo porcine disc degeneration model. Eur. Spine J..

[70] Koo Y.W., Lim C.S., Darai A., Lee J., Kim W., Han I., Kim G.H. (2023). Shape-memory collagen scaffold combined with hyaluronic acid for repairing intervertebral disc. Biomater. Res..

[71] Luo J., Darai A., Pongkulapa T., Conley B., Yang L., Han I., Lee K.B. (2023). Injectable bioorthogonal hydrogel (BIOGEL) accelerates tissue regeneration in degenerated intervertebral discs. Bioact. Mater..

[72] Panebianco C.J., Constant C., Vernengo A.J., Nehrbass D., Gehweiler D., DiStefano T.J., Martin J., Alpert D.J., Chaudhary S.B., Hecht A.C. (2023). Combining adhesive and nonadhesive injectable hydrogels for intervertebral disc repair in an ovine discectomy model. JOR Spine.

[73] Woiciechowsky C., Abbushi A., Zenclussen M.L., Casalis P., Krüger J.P., Freymann U., Endres M., Kaps C., Kaps C. (2014). Regeneration of nucleus pulposus tissue in an ovine intervertebral disc degeneration model by cell-free resorbable polymer scaffolds. J. Tissue Eng. Regen. Med..

[74] Choi M.H., Blanco A., Stealey S., Duan X., Case N., Sell S.A., Rai M.F., Zustiak S.P. (2020). Micro-clotting of platelet-rich plasma upon loading in hydrogel microspheres leads to prolonged protein release and slower microsphere degradation. Polymers.

[75] Nagae M., Ikeda T., Mikami Y., Hase H., Ozawa H., Matsuda K.I., Sakamoto H., Tabata Y., Kawata M., Kubo T. (2007). Intervertebral disc regeneration using platelet-rich plasma and biodegradable gelatin hydrogel microspheres. Tissue Eng..

[76] Sawamura K., Ikeda T., Nagae M., Okamoto S., Mikami Y., Hase H., Ikoma K., Yamada T., Sakamoto H., Matsuda K. (2009). Characterization of in vivo effects of platelet-rich plasma and biodegradable gelatin hydrogel microspheres on degenerated intervertebral discs. Tissue Eng. Part. A.

[77] Kumar H., Ha D.H., Lee E.J., Park J.H., Shim J.H., Ahn T.K., Kim K.T., Ropper A.E., Sohn S., Kim C.H. (2017). Safety and tolerability of intradiscal implantation of combined autologous adipose-derived mesenchymal stem cells and hyaluronic acid in patients with chronic discogenic low back pain: 1-year follow-up of a phase I study. Stem Cell Res. Ther..

[78] Zhou S., Schmelz A., Seufferlein T., Li Y., Zhao J., Bachem M.G. (2004). Molecular mechanisms of low intensity pulsed ultrasound in human skin fibroblasts. J. Biol. Chem..

[79] Pounder N.M., Harrison A.J. (2008). Low intensity pulsed ultrasound for fracture healing: A review of the clinical evidence and the associated biological mechanism of action. Ultrasonics.

[80] Iwabuchi S., Ito M., Hata J., Chikanishi T., Azuma Y., Haro H. (2005). In vitro evaluation of low-intensity pulsed ultrasound in herniated disc resorption. Biomaterials.

[81] Iwabuchi S., Ito M., Chikanishi T., Azuma Y., Haro H. (2008). Role of the tumor necrosis factor-α, cyclooxygenase-2, prostaglandin E2, and effect of low-intensity pulsed ultrasound in an in vitro herniated disc resorption model. J. Orthop. Res..

[82] Yu P., Mao F., Chen J., Ma X., Dai Y., Liu G., Dai F., Liu J. (2022). Characteristics and mechanisms of resorption in lumbar disc herniation. Arthritis Res. Ther..

[83] Zhang X.J., Hu Z., Hao J., Shen J. (2016). Low intensity pulsed ultrasound promotes the extracellular matrix synthesis of degenerative human nucleus pulposus cells through FAK/PI3K/Akt pathway. Spine.

[84] Tanveer F., Arslan S.A., Darain H., Ahmad A., Gilani S.A., Hanif A. (2022). Effects of low-intensity pulsed ultrasound on pain and functional disability in patients with early-stage lumbar spondylolysis: A randomized controlled trial. J. Bodyw. Mov. Ther..

[85] Yilmaz M., Tarakci D., Tarakci E. (2020). Comparison of high-intensity laser therapy and combination of ultrasound treatment and transcutaneous nerve stimulation on cervical pain associated with cervical disc herniation: A randomized trial. Complement. Ther. Med..

[86] Rubira A.P.F.A., Rubira M.C., Rubira L.A., Comachio J., Magalhães M.O., Marques A.P. (2019). Comparison of the effects of low-level laser and pulsed and continuous ultrasound on pain and physical disability in chronic non-specific low back pain: A randomized controlled clinical trial. Adv. Rheumatol..

[87] Boyraz I., Yildiz A., Koc B., Sarman H. (2015). Comparison of high-intensity laser therapy and ultrasound treatment in the patients with lumbar discopathy. BioMed Res. Int..

